# Olfactory Nerve Transection Transiently Activates Olfactory Ensheathing Cells in *Xenopus laevis* Larvae

**DOI:** 10.1111/ejn.70211

**Published:** 2025-08-04

**Authors:** Melina Kahl, Lukas Weiss, Joshua Walter, Thomas Hassenklöver, Ivan Manzini

**Affiliations:** ^1^ Institute of Animal Physiology, Department of Animal Physiology and Molecular Biomedicine Justus‐Liebig‐University Giessen Giessen Germany; ^2^ Department of Ecology and Evolutionary Biology Princeton University Princeton NJ USA

**Keywords:** axonal guidance, degeneration, injury, non‐neuronal cells, olfactory system, regeneration, ribosomal protein S6

## Abstract

Olfactory ensheathing cells (OECs) play a crucial role in supporting the continuous turnover of olfactory receptor neurons by promoting axon growth and targeting. While OECs have been extensively studied in mammals for their potential in treating nerve injuries, little is known about these cells in non‐mammalian vertebrates. We identified and characterized the morphology of OECs, fibroblasts, and macrophages in the olfactory system of *Xenopus laevis* larvae. Additionally, we used antibodies against phosphorylated ribosomal protein S6 (p‐rpS6) to visualize the activation of non‐neuronal cells in the olfactory nerve (ON) after transection. Various non‐neuronal cells in the ON, including OECs, fibroblasts, and macrophages, showed a transient increase in the p‐rpS6 signal following transection. Our study provides the first description of non‐neuronal cells populating the ON of larval *X. laevis*, and it suggests that rpS6 phosphorylation in these cells may be essential after injury and potentially supports regeneration of the ON. These findings contribute to a broader understanding of OECs and their role in nerve regeneration across species.

## Graphical abstract

- The olfactory nerve of larval *Xenopus* is populated by olfactory ensheathing cells, fibroblasts, and macrophages.
- Olfactory nerve transection leads to activation of non‐neuronal cells.
- Activated non‐neuronal cells of the olfactory nerve may support regenerative processes.

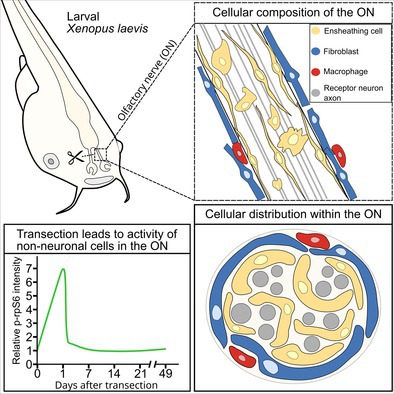

**Abbreviations**
OB: olfactory bulb
OE: olfactory epithelium
OECs: olfactory ensheathing cells
ON: olfactory nerve
ORNs: olfactory receptor neurons
p‐rpS6: phosphorylated ribosomal protein S6

## 1 Introduction

The olfactory system in vertebrates exhibits a remarkable capacity for regeneration, particularly in the replacement of olfactory receptor neurons (ORNs; Astic and Saucier 2001; Schwob 2002; Manzini 2015). When ORNs reach the end of their lifespan or if they are damaged, e.g., by exposure to toxins, infections, or injury, they undergo apoptosis and/or necrosis and are replaced by new neurons (Deckner et al. 1997; Astic and Saucier 2001; Brenneman et al. 2002; Schwob 2002; Manzini 2015; Hawkins et al. 2017; Werner and Nies 2018). New ORNs differentiate from a heterogeneous population of neuronal stem cells located in basal layers of the olfactory epithelium (OE; Schwob 2002; Ekberg et al. 2012; Manzini 2015) and subsequently extend unmyelinated axons to synaptically connect with projection neurons and interneurons of the olfactory bulb (OB) in the anterior telencephalon (Moreno‐Flores et al. 2002; Manzini et al. 2022).

The olfactory nerve (ON) is a critical component of the olfactory system, composed of bundles of ORN axons and several populations of non‐neuronal cells, including olfactory ensheathing cells (OECs), fibroblasts, and macrophages. OECs constitute a heterogeneous class of molecularly and functionally diverse glial cells that ensheathe bundles of ORN axons along the ON (Doucette 1993; Sonigra et al. 1999; Chuah and West 2002; Pellitteri et al. 2010; Su and He 2010; Higginson and Barnett 2011; Ekberg et al. 2012; Ekberg and St John 2014; Manzini et al. 2022). Axon bundles of ORNs are also surrounded by fibroblasts, which form a perineurium‐like structure (Li et al. 1998, 2005; Wright et al. 2020). In addition, the ON is populated by a small number of macrophages (Li et al. 1998, 2005; Wright et al. 2020).

The unambiguous identification of OECs is inherently difficult. In mammals, OECs have been shown to express, among others, vimentin, glial fibrillary acidic protein (GFAP), the calcium‐binding protein S100β, neural cell adhesion molecules (NCAMs), p75 neurotrophin receptors (p75NTR), neuropeptide Y, and several axon guidance molecules (Barnett and Chang 2004; Pellitteri et al. 2010; Higginson and Barnett 2011; Ekberg et al. 2012; Rawji et al. 2013; Oprych et al. 2017). Among those, particularly vimentin, a major intermediate filament protein (Ivaska et al. 2007), has been a helpful marker of OECs (Franceschini and Barnett 1996; Schwob et al. 1986; Ramón‐Cueto and Avila 1998; Au and Roskams 2003; Lazzari et al. 2013). The transcription factor s*ox10*, on the other hand, has been shown to label developing OECs in different vertebrate taxa (Barraud et al. 2010, 2013; Forni et al. 2011; Pingault et al. 2013; Oprych et al. 2017; Perera et al. 2020). None of these markers, however, is suitable for identifying all types of OECs. Fibronectin, an essential element of the extracellular matrix, is known to be expressed in fibroblasts (Ibanez et al. 2007) and has been used to mark fibroblasts in the mammalian ON (Li et al. 2008; Oprych et al. 2017). Macrophage‐expressed gene 1 (*mpeg1*), which encodes the pore‐forming protein perforin‐2, is evolutionarily conserved and commonly expressed in macrophages, making it a reliable marker for macrophage identification (Ellett et al. 2011; Bayly‐Jones et al. 2020; Ferrero et al. 2020).

Among the non‐neuronal cells of the ON, most information is available about OECs. While initially believed to originate from the olfactory placode, OECs are now widely accepted to derive from the neural crest (Chuah and Au 1991; Valverde et al. 1992; Barraud et al. 2010; Forni et al. 2011; Katoh et al. 2011; Nakajima et al. 2013; Denaro et al. 2022). The promotion of continuous growth and the targeted pathfinding of ORN axons and the phagocytosis of axonal debris within the ON are undoubtedly the key functions of OECs (Doucette 1993; Key and St John 2002; Pellitteri et al. 2010; Su and He 2010; Ekberg et al. 2012; Su et al. 2013; Ekberg and St John 2014; Nazareth et al. 2015; Barton et al. 2017; Denaro et al. 2022; Denaro et al. 2022; Manzini et al. 2022; Zhang et al. 2023). Additionally, OECs are involved in regulating the immune response within the ON (Chen et al. 2025).

OECs have been shown to support neural repair in the olfactory system and, after implantation, also in other regions of the nervous system (Su and He 2010; Ekberg et al. 2012; Roet and Verhaagen 2014). Their potential to promote spinal cord regeneration has been well studied (Raisman 2001; Barton et al. 2017; Huang et al. 2024), showing promising but still inconsistent outcomes (Mackay‐Sim 2005; Ekberg et al. 2012; Yao et al. 2018). The underlying molecular mechanisms, i.e., the secretion and regeneration‐supporting functions of adhesion molecules, extracellular matrix proteins, and growth factors (Bailey et al. 1993; Pellitteri et al. 2010; Barton et al. 2017; Russo et al. 2020), are not yet fully elucidated.

While extensive research has been conducted on OECs in mammals, information about OECs in non‐mammalian vertebrates is very limited (e.g., see Quintana‐Urzainqui et al. 2014; Docampo‐Seara et al. 2022: catshark; Kreutzberg and Gross 1977; Lazzari et al. 2013, 2014: teleosts; Daston et al. 1990; Burd 1991, 1992; Huang et al. 2005; Lazzari et al. 2016: adult and metamorphic amphibians).

Larvae of the African clawed frog *Xenopus laevis* are particularly suitable for studying injury‐mediated neuronal regeneration processes and are widely used in regenerative biology (Manzini 2015; Cervino et al. 2017; Hawkins et al. 2017; Hawkins et al. 2024; Terni et al. 2017; Lara et al. 2024). Nevertheless, the morphology and functions of OECs in *Xenopus* remain largely unclear. The present study aims to identify and describe OECs and other non‐neuronal cells in the larval *Xenopus* ON using transgenic animal lines, immunohistochemistry, and single‐cell electroporations. In addition, we describe activity changes in non‐neuronal cells of the ON, including OECs, that occur after injury of the olfactory system.

## 2 Materials and Methods

### 2.1 Animals

To obtain our results, we used wild‐type, albino, and transgenic *sox10*‐GFP [Xla.Tg (sox10:GFP)^Jpsj^] and *mpeg1*‐GFP [Xla.Tg (Dre.mpeg1:eGFP;cryga:mCherry)^Amaya^] (xenopusresource.org) larvae of *X. laevis* (both sexes). The larvae were maintained and raised in the breeding colony at the animal facility of the Institute of Animal Physiology at Justus Liebig University of Giessen. Premetamorphic larvae with well‐developed olfactory systems, ranging from developmental stages 48 to 52, were used (staged after Nieuwkoop and Faber 1994). The animals were kept in water tanks (7.5 L) at a temperature of 19°C–22°C (pH 7.8) and fed with algae (Hobby, Mikrozell, Dohse Aquaristik GmbH und Co. KG).

### 2.2 ON Transection

As an injury model for neuronal damage in the olfactory system, we unilaterally transected an ON of larval *X. laevis* (also see Hawkins et al. 2017, Hawkins et al. 2024). The larvae were anesthetized in 0.02% MS‐222 (ethyl 3‐aminobenzoate methanesulfonate; Sigma‐Aldrich). The ON was then transected with fine scissors without damaging the surrounding tissue. The wound was then closed with tissue adhesive (Histoacryl L; Braun). After this procedure, the animals were transferred to a beaker with fresh tap water for recovery. At different time intervals after injury, transected animals were again anesthetized in 0.02% MS‐222 and killed by severing the brain and spinal cord with a scalpel. Subsequent experiments were performed on excised tissue blocks containing the OE, ONs, and OB.

### 2.3 ORN Labeling via Electroporation

To visualize ORN axons in the ON, fluorophore‐coupled dextran (Alexa 488 or Alexa 594; 10.000 MW, Molecular Probes, Thermo Fisher Scientific) was introduced into ORNs via electroporation (for details, see Hassenklöver and Manzini 2014). *X. laevis* larvae were anesthetized in 0.02% MS‐222, and dye crystals of dextran were introduced and dissolved into both nasal cavities. Two platinum electrodes were placed, one inside the nasal cavity and the other at the tissue surrounding the nostril. The electrodes were connected to a voltage pulse generator (ELP‐01D; npi Electronics), and six pulses (15 V, 25 ms duration at 2 Hz) with alternating polarity were applied (also see Weiss et al. 2018). The procedure was repeated with the other nasal cavity. After electroporation, the animals were transferred to a beaker filled with fresh tap water for recovery. The right ON was transected 3 or 4 days after electroporation (see section above). The animals were sacrificed at different time points after ON transection (1 h, 3 h, 6 h, 12 h, 24 h, 48 h, 72 h, 1 week, 2 weeks, 3 weeks, and 7 weeks) and further processed. To visualize the regenerated ON at later time points after transection, the ONs of the animals were first transected (see section above) and then electroporated 3 days before they were killed and further processed.

### 2.4 Whole Mount Preparation and Immunohistochemistry of the Olfactory System

To label different cell types and to visualize changes in the ON and OB after unilateral ON transection, we performed immunolabeling on tissue slices of the olfactory system. Animals were anesthetized (as described above) and killed by severing the brain at its transition to the spinal cord with a scalpel. Tissue blocks with complete olfactory systems containing the OE, ONs, and OB were cut out with fine scissors and fixed in 4% Roti‐Histofix (Roth, pH 7) for 1 h at room temperature on a shaker in an Eppendorf tube. Subsequently, the tissue blocks were washed in 0.1 M phosphate buffer saline (PBS), glued onto a vibratome stage (VT 1200S, Leica), and cut horizontally into 300 μm thick slices. Tissue slices were permeabilized with PBST (PBS containing 0.2% Triton X100; Carl Roth), and non‐specific binding was blocked with 2% normal goat serum (NGS; MP Biomedicals) for 1 h. Slices were incubated over two nights at 4°C with the following primary antibodies: anti‐vimentin (14h7, RRID: AB_528507, monoclonal, derived from mouse, Developmental Studies Hybridoma Bank); anti‐fibronectin (MA5‐11981, RRID: AB_10982280, monoclonal, derived from mouse, Thermo Fisher Scientific); anti‐phospho‐S6 ribosomal protein (Ser235/236) (4858S, RRID: AB_916156, monoclonal, derived from rabbit, Cell Signalling Technology); anti‐HuC/HuD (HuC/D) (A‐21271, RRID: AB_221448, monoclonal, derived from mouse, Thermo Fisher Scientific). Green fluorescent protein (GFP) signals of transgenic animals were enhanced with anti‐GFP (ab1218, RRID: AB_298911, monoclonal, derived from mouse; Abcam).

Primary antibodies were diluted in 2% NGS/PBST (anti‐vimentin, anti‐phospho‐S6 ribosomal protein [Ser235/236], anti‐HuC/D: 1:250, anti‐fibronectin: 1:100, anti‐GFP: 1:50) and washed off with PBS after the incubation period. Subsequently, Alexa 488 or 594‐conjugated goat anti‐mouse or anti‐rabbit secondary antibodies (Invitrogen, Thermo Fisher Scientific) were applied at a dilution of 1:250 in 2% NGS/PBS over one night. The secondary antibodies were washed off with PBS. To stain cell nuclei, the tissue samples were incubated for 20 min in 10 μg/mL propidium iodide (Molecular Probes, Thermo Fisher Scientific). After repeated washing with PBS, the slices were transferred into a small petri dish filled with 500 μL PBS and stabilized with a platinum frame stringed with nylon threads.

### 2.5 Sparse Cell Electroporation to Label Individual Cells of the ON

To examine and visualize the morphology of individual cells in the ON, cells of the ON were labeled using single‐cell electroporation. Tissue blocks, containing the entire olfactory system, were cut out with fine scissors (as described above) and transferred into frog saline ringer (98 mM NaCl, 2 mM KCl, 1 mM CaCl_2_, 2 mM MgCl_2_, 5 mM glucose, 5 mM Natrium‐pyruvate, 10 mM HEPES; 230 mOsmol/l, pH 7.8). The palatial tissue covering the ventral side of the ON and OB was removed. The samples were transferred, with the ventral surface facing up, into a small petri dish with a recess in the middle, filled with 500 μL frog saline ringer, and stabilized with a platinum frame stringed with nylon threads. The samples were then placed under a microscope with fluorescent illumination.

Electroporation micropipettes were produced from borosilicate glass capillaries (inner diameter 0.86 mm, outer diameter 1.5 mm, length 10 cm, Hilgenberg) with a horizontal micropipette puller (Model P‐1000, Sutter Instruments). The micropipettes were filled with 3 μL Alexa 488 or Alexa 594 dextran solution (10 kDa MW, 3 mM in frog saline ringer, Life Technologies, Invitrogen, Thermo Fisher Scientific) and mounted on the head stage of an Axoporator 800 A (Molecular Devices). This head stage includes a silver wire electrode coated with silver chloride. A reference electrode connected to the Axoporator was placed near the tissue block to close the electrical circuit.

After positioning the micropipette in the frog saline ringer (see above), it was directed toward the ONs using a micromanipulator (PatchStar, Scientifica). When the pipette was in contact with a cell of interest, a train of positive square voltage pulses (50 V, 300 μs, 300 Hz for 500 ms) was triggered to transfer the charged dye along an electric field into a single cell (also see Weiss et al. 2018). Samples were fixed in 4% Roti‐Histofix (Roth, pH 7) for 1 h at room temperature. The tissue blocks were then washed in 0.1 M PBS, and nuclear staining was performed with propidium iodide (see above).

### 2.6 Multiphoton Imaging

The ONs and the OB were observed using an upright multi‐photon microscope (A1R‐MP, Nikon). Images were acquired with an excitation laser wavelength of 780 nm as virtual stacks with a z‐resolution of 1–3 μm. Fluorescence emission was detected with three different detectors (blue 400–492 nm, green 500–550 nm, and red 601–657 nm).

### 2.7 Image and Data Processing

The brightness and contrast of the image stacks obtained from scans of the multi‐photon microscope were adjusted using the image processing software ImageJ (Schindelin et al. 2012). When excited by the multiphoton laser, pigments showed strong autofluorescence and were visible as supersaturated image areas. The blue emission detector (400–492 nm) was used to identify autofluorescence. As we never used samples with blue marker fluorophores, no fluorescence was expected in the blue channel. This allowed mathematical subtraction of autofluorescence (blue detector signal) from the other channels. The volume viewer in ImageJ was used to create virtual cross‐sections of the ONs. A median filter with a radius of 0.5 pixels was applied to all planes of the image stacks to reduce background noise. After processing, the images were arranged and annotated with Inkscape (inkscape.org).

In the present study, we used immunohistochemistry against phosphorylated ribosomal protein S6 (p‐rpS6) as a proxy for cellular responses after ON transection and during regenerative processes (Knight et al. 2012; Biever et al. 2015; Ring et al. 2023). To quantify cellular activity based on p‐rpS6 stainings in unilaterally ON‐transected animals, ON/OB hemisphere samples of the same animal were acquired with identical multi‐photon microscope settings (gain, laser power, and scan parameters). Supersaturated areas in image stacks were mathematically subtracted as described above. Image stacks were individually cropped in the y‐direction to exclude cellular p‐rpS6 signals from the OB. The cropping range was determined manually, and the final image size was similar on the transected and non‐transected sides of the animal. A Gaussian filter (σ = 35) was applied to all planes to estimate the background signal, which was then subtracted from the image stacks. Then, all image planes were median‐filtered with a three‐pixel radius. To measure the effect of ON transection on the p‐rpS6 signal, we calculated a ratio of the cumulative p‐rpS6 signal intensity between transected and non‐transected ON of the same animal (transected/non‐transected). Data analysis was performed using custom‐written scripts in Python 3 in Jupyter Notebook (code available under https://doi.org/10.22029/jlupub‐19833).

### 2.8 Statistical Analysis

The results are presented as mean ± standard deviation unless otherwise stated. Statistical significance was determined by the Kruskal–Wallis test, followed by Dunn's multiple comparison post hoc test. We compensated for the family‐wise error rate using the Bonferroni‐Holm method.

## 3 Results

### 3.1 The ON Is Populated by Ensheathing Cells, Fibroblasts, and Macrophages

The cellular composition of the ON of *X. laevis* is still largely unknown. To visualize OECs, fibroblasts, and macrophages, we performed immunolabeling on tissue slices of the olfactory system of wild‐type and transgenic animals.

**FIGURE 1:**
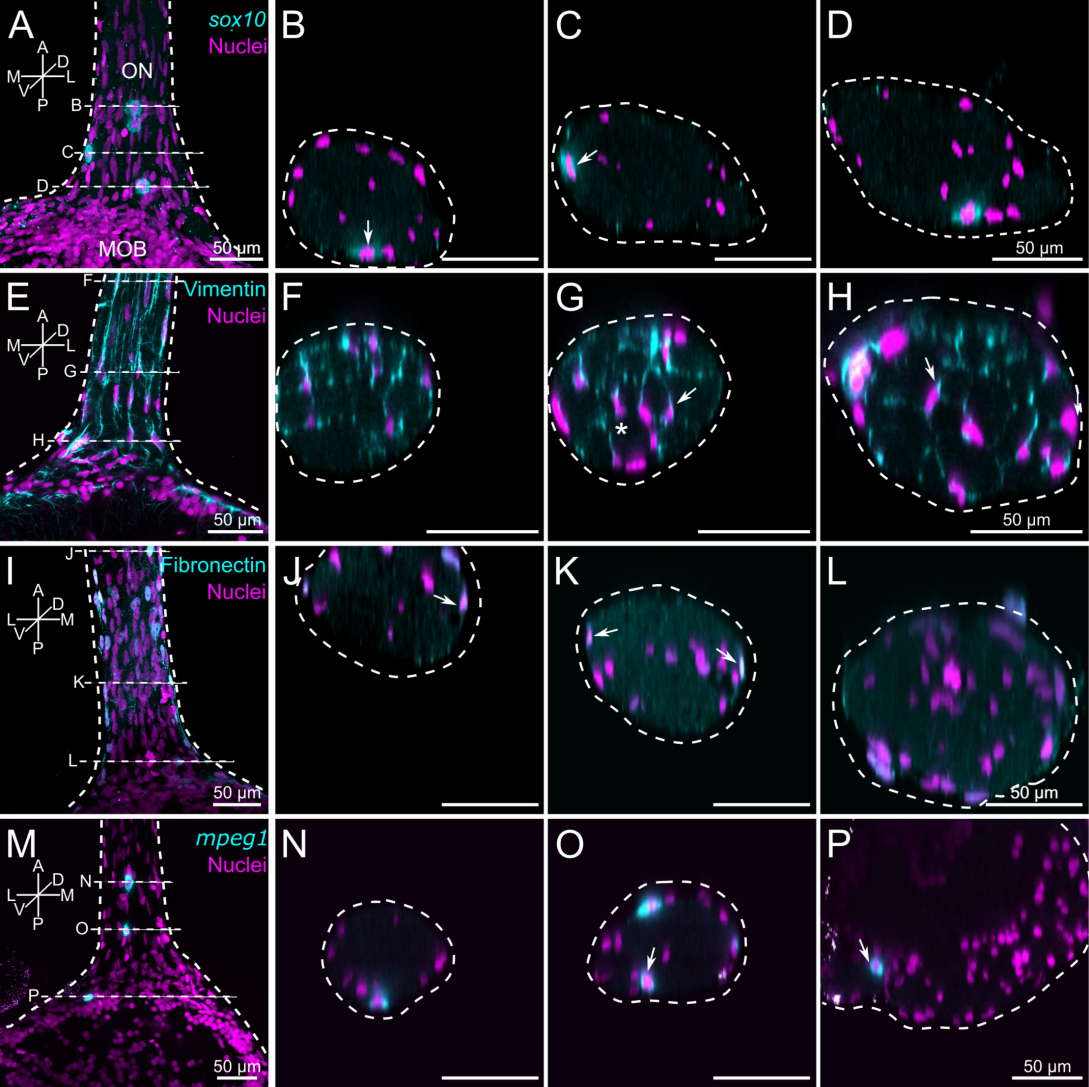
Identification of non‐neuronal cells within the olfactory nerve. (A) *sox10*‐positive cells (cyan) within the ON. (B–D) Orthogonal projections of the same image stack as in A, visualizing sections at three different locations on the anterior–posterior axis of the ON. (B) anterior; (C) intermediate; and (D) posterior. White arrows point at some *sox10*‐positive cells located on the surface of the ON. (E) Vimentin‐positive cells (cyan) with a spindle‐like bipolar morphology are found throughout the ON and the nerve layer of the OB. (F–H) Orthogonal projections of the same image stack as in E, visualizing sections through the ON. (F) anterior; (G) intermediate; and (H) posterior, close to the OB. White arrows point at some vimentin‐positive cells. The asterisk indicates vimentin‐positive cells surrounding unstained areas within the ON. (I) Fibronectin‐positive cells (cyan) have a long, flat, and oval‐shaped morphology and are localized on the surface of the ON. (J‐L) Orthogonal projections of the same image stack as in I, visualizing sections through the ON. (J) anterior; (K) intermediate; and (L) posterior, close to the OB. Fibronectin‐positive cells encase the whole ON (white arrows). (M) *mpeg1*‐positive macrophages (cyan) of different sizes have a round to oval‐shaped morphology and are predominantly located on the surface of the ON and within the OB. (N–P) Orthogonal projections of the same image stack as in M, visualizing sections through the ON. (N) anterior; (O) intermediate; and (P) posterior, close to the OB. White arrows point at macrophages. Similar results were obtained in all animals investigated (*sox10*: 4 animals; vimentin: 6 animals; fibronectin: 10 animals; *mpeg1*: 8 animals). Cell nuclei were labeled with propidium iodide (PI, magenta). A, anterior; D, dorsal; L, lateral; M, medial; MOB, main olfactory bulb; ON, olfactory nerve; P, posterior; V, ventral.

Using a *sox10*‐GFP transgenic *Xenopus* line, we identified a small number of round‐shaped cells primarily located near the surface of the ON (Figure 1A–D). The localization, number, and morphology of these cells are not consistent with descriptions of mature OECs. These cells could, however, possibly be developing immature OECs. Immunolabeling against vimentin stained cells in the ON and the nerve layer of the OB. These cells exhibited a fusiform bipolar morphology characterized by thin extensions originating from their centrally located nuclei (Figure 1E). In cross‐sections of the ON, we found that the vimentin‐positive cells were present throughout the whole nerve (Figure 1F–H). On average, each ON contained 316 ± 33 vimentin‐positive cells (*n* = 3 ONs). These cells consistently surrounded unstained areas of the ON, containing ORN‐axons (Figure 1G, asterisk; supplementary Figure S1). The localization, number, and morphology of these cells are consistent with earlier descriptions of OECs in different species (Doucette 1993; Sonigra et al. 1999; Chuah and West 2002; Pellitteri et al. 2010; Su and He 2010; Higginson and Barnett 2011; Ekberg et al. 2012). An antibody against fibronectin labeled spindle‐like and oval‐shaped cells, which were located on the surface of the ON (Figure 1 J‐K). These cells encased the ON along its entire length (Figure 1I–L). On average, each ON contained 37 ± 9 fibronectin‐positive cells (*n* = 3 ONs). The localization, number, and morphology of these cells are consistent with earlier descriptions of fibroblasts in different species (Li et al. 2008; Oprych et al. 2017). Using a *mpeg1*‐GFP transgenic *Xenopus* line, we identified a small number of round to oval‐shaped macrophages mainly located on the surface of the ON and in some cases in the nerve layer of the OB (Figure 1M–P). On average, each ON contained 4 ± 1 *mpeg1*‐GFP‐positive cells (*n* = 3 ONs).

Together, the location within the ON, morphology, and expression pattern of specific markers allowed us to identify at least three distinct cell types: vimentin‐positive putative OECs, fibronectin‐positive putative fibroblasts, and *mpeg1*‐GFP‐positive putative macrophages. Among all cells stained with the three adopted markers, ~89% were vimentin‐positive, ~10% were fibronectin‐positive, and ~1% were *mpeg1*‐GFP‐positive.

### 3.2 Electroporated Cells of the ON Can Be Classified Into Two Main Morphological Types

We performed single‐cell electroporations to visualize the morphology of individual non‐neuronal cells of the ON. Electroporation of fluorescently‐tagged dextran labeled the cytosol, including the various appendages of cells.

**FIGURE 2:**
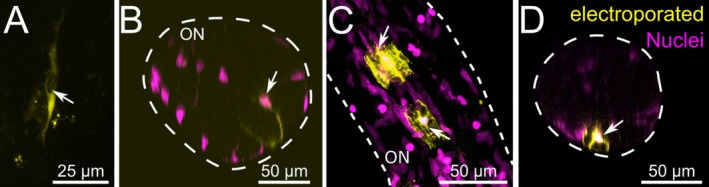
Electroporated non‐neuronal cells in the olfactory nerve can be grouped into two main categories. (A) Example of a fusiform bipolar cell situated in deeper layers of the ON with two extensions (yellow) originating from the centrally located nucleus (not stained; arrow). (B) Orthogonal projections of another ON. The arrow indicates the nucleus (magenta) of an electroporated fusiform bipolar cell. The extension of this cell (yellow) forms a circle around an empty area of the ON. (C) Two electroporated cells with a flat, sheet‐like morphology located on the surface of the ON (yellow). Arrows label protrusions originating from the centrally located nuclei (magenta). (D) Orthogonal projections of another ON. The arrow indicates an electroporated cell with a flat, sheet‐like morphology (yellow). Cell nuclei were labeled with propidium iodide (PI, magenta). ON, olfactory nerve.

We performed single‐cell electroporations in 14 larvae of *X. laevis* and stained 74 individual cells within the ON. The 74 labeled cells could be categorized into two main morphological types (Figure 2). In deeper layers of the ON, the cells exhibited a fusiform bipolar morphology featuring two extensions originating from the centrally located nucleus (nucleus not stained in Figure 2A). The length of the cell shown in Figure 2A is approximately 70 μm. Figure 2B shows an orthogonal projection of another ON with an electroporated fusiform bipolar cell. Of the 74 individual stained cells, 49 had fusiform bipolar morphologies similar to those shown in Figure 2A,B. In more superficial layers of the ON, the electroporated cells had a considerably different morphology (Figure 2C,D). These cells (yellow) had a more compact, flat, and sheet‐like morphology, with several protrusions originating from the centrally located nuclei (magenta). The length of the cells shown in Figure 2C is approximately 30 μm. Of the 74 individual stained cells, 25 had morphologies like those shown in Figure 2C,D.

In conclusion, non‐neuronal cells labeled via single‐cell electroporation in the ON can be grouped into two main types: fusiform bipolar and flat, sheet‐like.

### 3.3 ON Transection Leads to Phosphorylation of rpS6 in Non‐Neuronal Cells in the ON

We performed immunolabelings of the olfactory system of larval *X. laevis* with an antibody against p‐rpS6 24 h after unilateral nerve transection.

**FIGURE 3:**
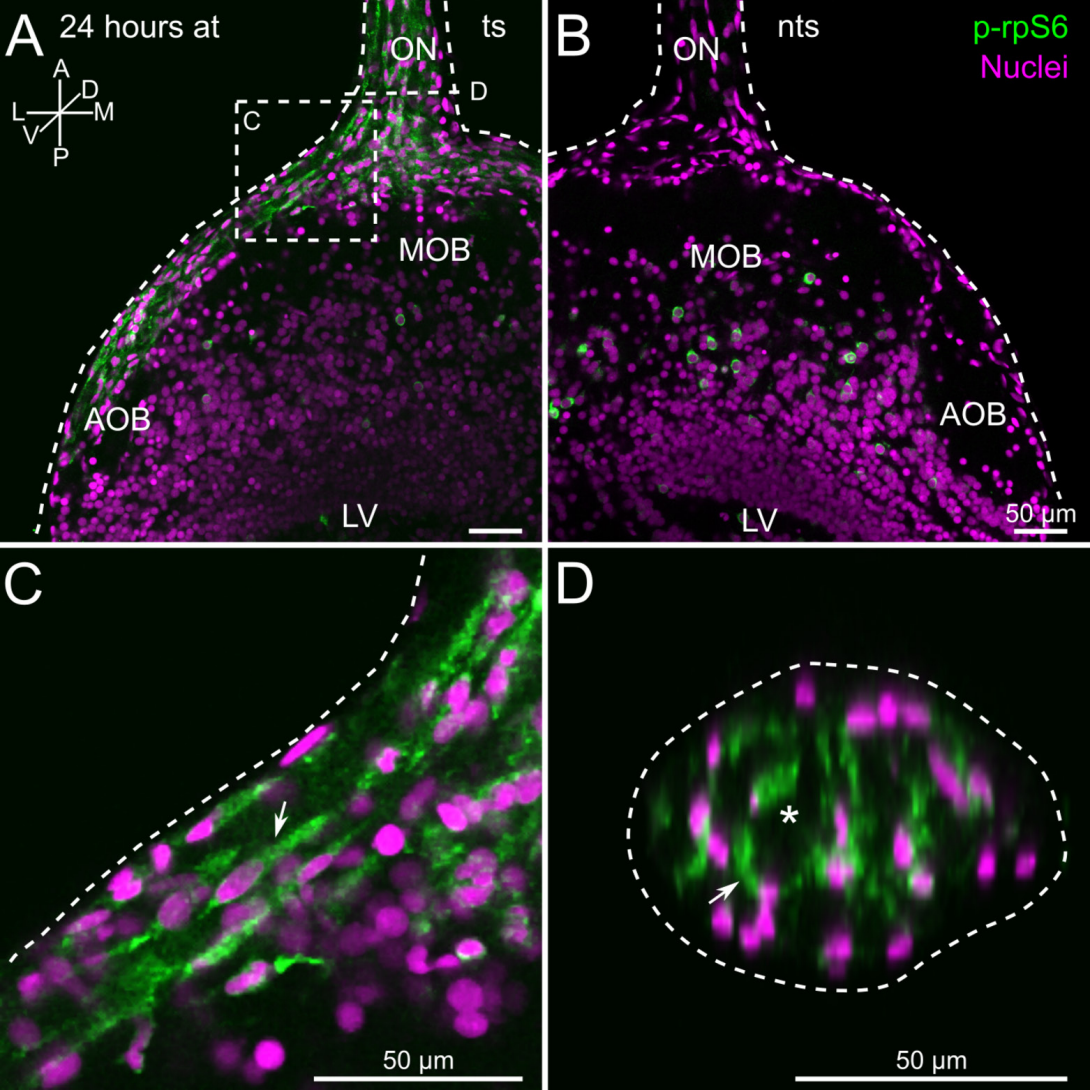
Olfactory nerve transection induces phosphorylation of rpS6 in non‐neuronal cells. (A and B) p‐rpS6 (green) labeled cells in the ON and OB. In the projection neuron layer of the OB, labeled cells are present on both the non‐transected and transected sides. In the nerve layer of the OB and the ON, stained cells are present only on the transected side. (C) Close‐up of the encircled area in (A). The p‐rpS6‐expressing cells in the ON have a fusiform bipolar morphology (white arrow). (D) Cross‐section through the nerve shown in A. Stained cells are present across the entire width of the ON (white arrow) and surround circular unstained gaps (white asterisk). Similar results were obtained in all animals investigated (*n* = 6). Cell nuclei were labeled with propidium iodide (PI, magenta). A, anterior; AOB, accessory olfactory bulb; at, after transection; D, dorsal; L, lateral; LV, lateral ventricle; M, medial; MOB, main olfactory bulb; nts, non‐transected side; ON, olfactory nerve; P, posterior; ts, transected side; V, ventral.

Expression of p‐rpS6 was observable in cells of the ON, the ON layer of the OB, the glomerular layer, and the projection neuron layer of the OB (Figure 3A,B). In the OB, labeled cells were present on both the non‐transected and transected sides. In the ON, on the other hand, p‐rpS6‐positive cells were mainly found on the transected side. Phosphorylated rpS6 evoked a strong cytoplasmic staining in cells with fusiform bipolar morphology (Figure 3C). Cell nuclei stained with propidium iodide were localized in the center of these cells (Figure 3C). Cross‐sectional rendering of the ON revealed that p‐rpS6‐positive cells were present throughout the whole width of the ON (Figure 3D). We regularly observed that these cells surrounded empty areas of the ON (Figure 3D, asterisks), which contained axons of ORNs (see supplementary Figure S1).

To distinguish the activated cell types within the ON, we performed immunohistochemistry using previously identified markers (see Figure 1) and an antibody against p‐rpS6.

**FIGURE 4:**
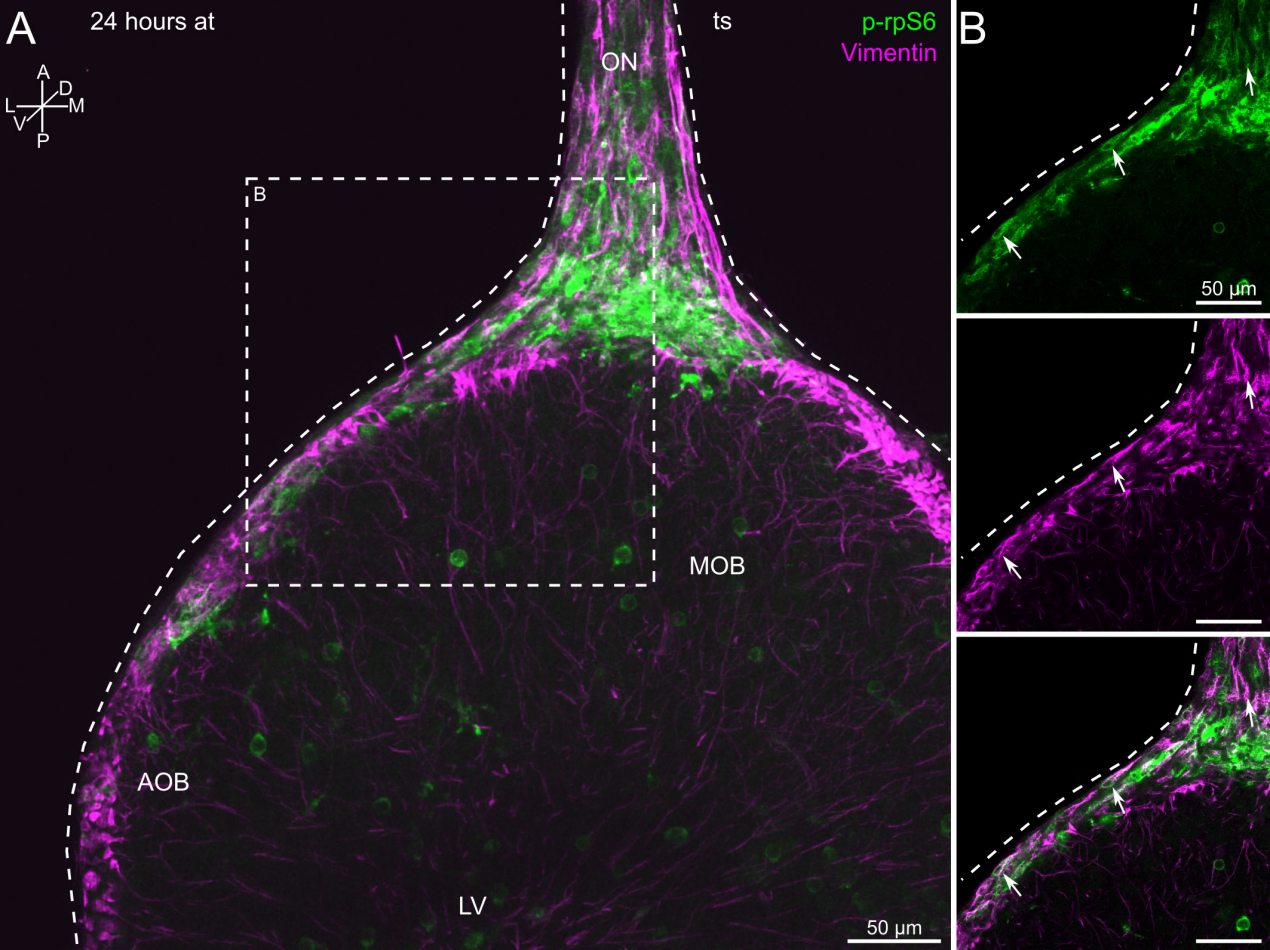
Vimentin and p‐rpS6 are co‐expressed in cells of transected olfactory nerves. (A) Vimentin (magenta) and p‐rpS6 (green) labeled cells populating the transected ON and the nerve layer of the OB. In addition, some ON and OB cells were labeled either by vimentin or p‐rpS6. (B) A close‐up of the area encircled in A shows the co‐localization of vimentin and p‐rpS6 in ON cells and their morphology in more detail. The white arrows indicate cells labeled by both markers (upper panel: p‐rpS6 channel; middle panel: vimentin channel; lower panel: overlay of both channels). Similar results were obtained in all animals investigated (*n* = 9). Note that the images in A and B result from individually recorded image stacks. A, anterior; AOB, accessory olfactory bulb; at, after transection; D, dorsal; L, lateral; LV, lateral ventricle; M, medial; MOB, main olfactory bulb; ON, olfactory nerve; P, posterior; ts, transected side; V, ventral.

Vimentin‐positive cells, most likely OECs (see also Figure 1), in the ON and the nerve layer of the OB also expressed p‐rpS6 (Figure 4A). A high number of double‐positive cells for both p‐rpS6 and vimentin could be observed (Figure 4B, arrows). The double‐positive cells had a fusiform bipolar morphology, indicating that most of these cells are OECs (Figure 4B). However, the number of vimentin‐positive cells was higher and more widespread than the p‐rpS6‐positive cells. This observation can, in part, be explained by the fact that the antibody against vimentin, in addition to cells of the ON, also stained a network of fibers spreading through the whole OB in both the non‐transected and transected sides (Figure 4A, Supplementary Figure S2). They were previously identified as processes of radial glial cells (see discussion).

**FIGURE 5:**
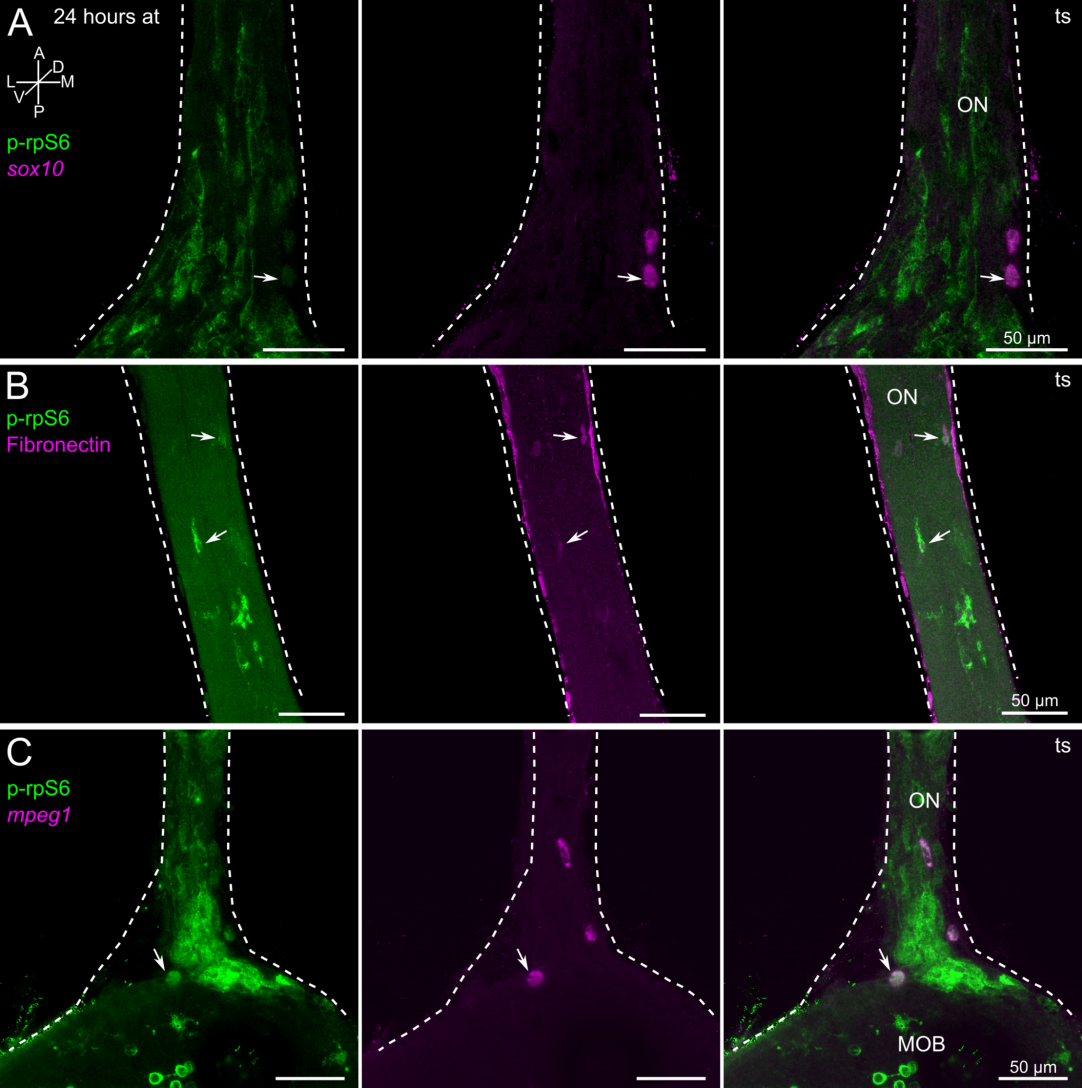
Non‐neuronal cells express the activity marker p‐rpS6 after olfactory nerve transection. (A) p‐rpS6 and *sox10*‐GFP are co‐localized in cells of transected ONs (arrows). (B) Co‐localization of fibronectin and p‐rpS6 in flat oval‐shaped cells of the transected ON (arrows). (C) p‐rpS6‐positive macrophages in the transected ON and the transition area between the ON and the OB (arrows). The left‐hand column shows the p‐rpS6 signal (green), the intermediate column shows the *sox10*, fibronectin, and *mpeg1* signal (magenta), and the right‐hand column shows merged images. Similar results were obtained in all animals investigated (*sox10*: 10 animals; fibronectin: 10 animals; *mpeg1*: 7 animals). A, anterior; at, after transection; D, dorsal; L, lateral; M, medial; MOB, main olfactory bulb; ON, olfactory nerve; p, posterior; ts, transected side; V, ventral.

Using transgenic *sox10*‐GFP *Xenopus* larvae, we identified several *sox10*‐positive cells in the ON also expressing p‐rpS6 (Figure 5A). The *sox10* and p‐rpS6 double‐positive cells had a round shape and were mainly localized near the surface of the ON (Figure 5A). Double‐staining with antibodies against fibronectin and p‐rpS6 double‐labeled several elongated cells with a spindle‐like morphology located on the surface of the ON, and some cells that were oval‐shaped (Figure 5B, arrows). These observations showed that only a few fibronectin‐positive fibroblasts were activated after transection and expressed p‐rpS6. Using the transgenic *mpeg1 Xenopus* larvae, we also observed that ON macrophages expressed p‐rpS6 after nerve transection. The macrophages were located on the surface of the ON and the transition area between the ON and the OB (Figure 5C, arrow).

In addition to the stainings in the ON and the nerve layer of the OB, p‐rpS6‐positive cells were also observed in the glomerular and mitral cell layers of the OB. The localization of the p‐rpS6‐positive cells in these layers suggested that these cells are projection neurons and/or periglomerular cells. The neuronal identity of these cells was confirmed by co‐labeling with HuC/HuD neuronal protein (HuC/D), known to label neuron‐specific ELAV/Hu RNA binding proteins (Wei and Lai 2022), and p‐rpS6 (Supplementary Figure S3).

Together, we found a high expression of p‐rpS6 in non‐neuronal cells of the ON 24 h after nerve transection. Several non‐neuronal ON cells, including numerous OECs, fibroblasts, and macrophages, were among the p‐rpS6‐positive cells. The expression of p‐rpS6 was strong in transected ONs and the ipsilateral nerve layer of the OB. We conclude that p‐rpS6 is an excellent marker for injury‐dependent activation of non‐neuronal cells in the olfactory system of larval *X. laevis* .

### 3.4 Non‐Neuronal Cells in the ONs Are Temporarily Activated Within 1–48 h After the Transection of the ON

To investigate the time course of activation of non‐neuronal ON cells, we unilaterally transected the ON of *X. laevis* larvae and examined the p‐rpS6 signal at different time points after transection. We calculated a ratio of the cumulative p‐rpS6 signal between the transected and non‐transected sides for each animal.

**FIGURE 6:**
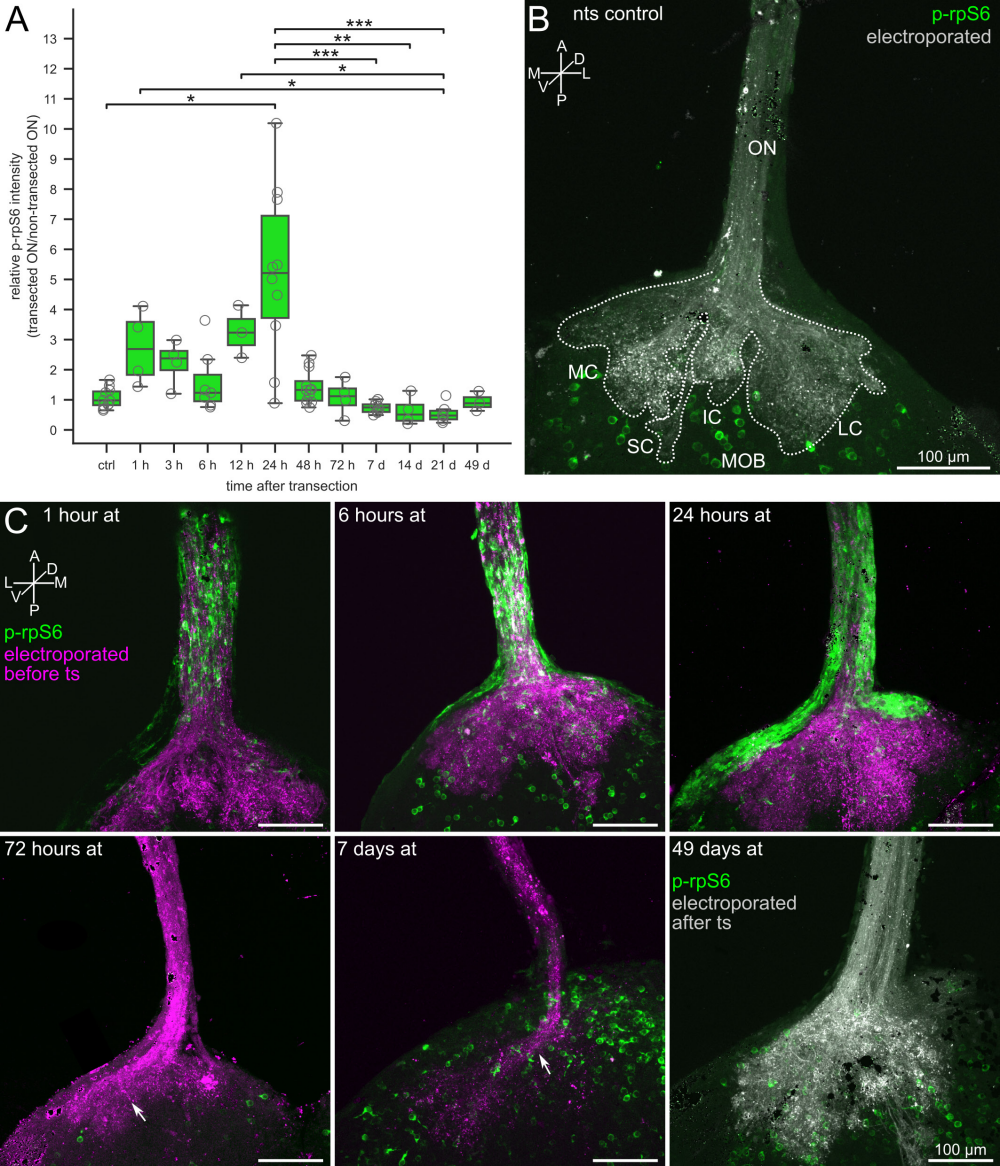
Non‐neuronal cells of the olfactory nerve feature transient phosphorylation of rpS6 after olfactory nerve transection. (A) Boxplot of p‐rpS6 immunohistochemically labeled cells at different time points after unilateral ON transection. A ratio of cumulative pixel intensities between the transected and non‐transected ON of the same animal was calculated to measure changes in activity. Individual data for each animal is marked with grey circles (ctrl, *n* = 11;1 h, *n* = 4; 3 h, *n* = 4; 6 h, *n* = 7; 12 h, *n* = 3; 24 h, *n* = 10; 48 h, *n* = 12; 72 h, *n* = 4; 7 d, *n* = 8; 14 d, *n* = 4; 21 d, *n* = 6; 49 d, *n* = 3). The black horizontal line indicates the median signal ratio of the animals in the same group. (B) Image of the ON and the OB of a non‐transected animal (white, axons of ORNs; green, p‐rpS6). The axons of ORNs were stained by nasal electroporation of fluorescent dextrans. The glomerular clusters are outlined with a dotted white line. Several p‐rpS6‐positive cells (green) were visible in the glomerular and the projection neuron layer of the OB. (C) Transection‐induced phosphorylation of rpS6 in non‐neuronal cells of the ON and nerve layer of the OB (different time points after transection). Axons of ORNs (magenta) were labeled by nasal electroporation of fluorescent dextrans before ON transection. The transection of the ON induced gradual axonal degradation in the OB. Seven weeks after ON transection, the reinnervation by ORN axons of the OB was reestablished. The axons of regenerated ORNs (white) were labeled by nasal electroporation of dextran 3 days before sample preparation and immunohistochemistry. A, anterior; AOB, accessory olfactory bulb; at, after transection; d, days; D, dorsal; Elp., electroporated; h, hours; IC, intermediate cluster; L, lateral; LC, lateral cluster; LV, lateral ventricle; M, medial; MC, medial cluster; MOB, main olfactory bulb; nts, non‐transected side; ON, olfactory nerve; P, posterior; SC, small cluster; V, ventral. Statistical significance was tested using the Kruskal‐Wallis test followed by Dunn's post hoc test with Bonferroni‐Holm correction (*, *p* < 0.05; **, *p* < 0.01; ***, *p* < 0.001).

In non‐transected control animals, the p‐rpS6 signal was equal in both ONs with a mean signal ratio of 1.0 ± 0.3. We investigated multiple time points, ranging from 1 h to 7 weeks, after ON transection, and the median p‐rpS6 signal ratio was statistically not equal across all samples (Figure 6A, Kruskal–Wallis, *p* = 2 * 10^−6^). ON transection led to a noticeable but not significant increase in p‐rpS6 signal on the transected side already after 1 h (Figure 6A–C). This trend was observable with continuously elevated p‐rpS6 signal ratios until 24 h after transection. After 24 h, the p‐rpS6 signal ratio was significantly increased to 5.2 ± 2.9 (*p* < 0.03). After 48 h, we observed a substantial decrease in the signal to a ratio of 1.4 ± 0.6. This trend continues with highly significant activity differences measured between 24 h and 7 days (*p* < 0.001), 14 days (*p* < 0.01), and 21 days (*p* < 0.001) after transection (Dunn's post hoc test with the Bonferroni–Holm correction).

In addition to the transient elevation of the p‐rpS6 signal, we also observed spatial dynamics of the signal localization (Figure 6C). One hour after the ON transection, the p‐rpS6 signal was mainly limited to the ON, and nearly no signal was found in the nerve layer of the OB. Six hours after transection, the signal was also visible in the nerve layer of the OB. Twenty‐four hours after transection, p‐rpS6‐positive cells were visible along the entire ON and within the nerve layer of the OB.

In addition, the connectivity of ORN axons and the morphology of glomeruli in the OB were observed by labeling ORN axons via electroporation. ON transection induced degradation of ORN axons. Over the first week after nerve transection, the OB area innervated by ORN axons gradually decreased on the transected side (Figure 6C; magenta axons). Seven days after nerve transection, most transected axons were already decomposed (Figure 6C; compare with Figure 6B, control side). Twenty‐one days after unilateral nerve transection, axons of newly formed ORNs (Figure 6C) had already extensively innervated the OB, and the reinnervation of the OB was widely completed after 49 days (Figure 6C, white axons).

In summary, non‐neuronal cells in the transected ON featured a transient increase in p‐rpS6, indicating that the activation of these cells was induced by ON transection. This activation begins within 1 h after the transection and reaches its peak after 24 h. Furthermore, we observed that the p‐rpS6 signal spreads from the ON to the OB within the first 24 h after transection. Within 7 days after ON transection, the p‐rpS6 signal returns to control conditions.

## 4 Discussion

In the present study, we characterized several classes of non‐neuronal cells populating the ON of larval *X. laevis* . We provide evidence that the ON houses OECs, fibroblasts, and macrophages. Furthermore, by employing the phosphorylation of rpS6 as a proxy for cellular activation, we show that several classes of non‐neuronal cells of the ON are transiently activated upon nerve injury. Possible physiological functions of the activation of these non‐neuronal cells are discussed.

### 4.1 Identification and Localization of Non‐Neuronal Cells Within the ON of Larval *X. laevis*

*Sox10* is known to be expressed in developing OECs in various vertebrate taxa (Barraud et al. 2010, 2013; Forni et al. 2011; Pingault et al. 2013; Oprych et al. 2017; Perera et al. 2020), and it is used as a marker for cells derived from the neural crest (Alkobtawi et al. 2018; Yang et al. 2020). Based on this information, we hypothesized that *sox10* could be expressed in OECs of *Xenopus* larvae. Using a *sox10*‐GFP *Xenopus* line, we identified a small number of *sox10*‐GFP positive cells with a round‐shaped morphology located within and on the surface of the ON (see Figures 1 and 5). The morphology, localization, and number of s*ox10*‐GFP positive cells indicate that these cells are not mature OECs nor other known non‐neuronal cells of the ON. We currently do not know the identity of these *sox10*‐GFP‐positive cells present in the ON of larval *Xenopus*. These cells may be developing, immature precursors on their way to becoming mature OECs.

Next, we employed an antibody against vimentin, a known component of the mammalian OEC intermediate filaments (Franceschini and Barnett 1996). In the ON, vimentin‐positive cells exhibited a long, fusiform bipolar morphology and ensheathed bundles of ORN axons throughout the ON. Thereby, vimentin‐positive structures resembled filamentous cytoskeletal elements (see Figure 1). Together, the characteristic vimentin staining is in line with the description of OEC in many vertebrate species (Doucette 1993; Sonigra et al. 1999; Chuah and West 2002; Pellitteri et al. 2010; Su and He 2010; Higginson and Barnett 2011; Ekberg et al. 2012; Manzini et al. 2022). It is thus very likely that most vimentin‐positive cells in the ON of larval *X. laevis* are indeed OECs. As vimentin is also expressed in fibroblasts (Cheng et al. 2016; Tallquist and Molkentin 2017; Sliogeryte and Gavara 2019), we can, however, not exclude that a subgroup of the vimentin‐positive cells represents fibroblasts. In addition, vimentin also stained many cells within the OB (see Figure 4 and Supplementary Figure S2). In *Xenopus*, these cells have already been identified as radial glia extending from the surface of the lateral ventricles to the surface of the OB (Nezlin et al. 2003; Huang et al. 2005).

To label fibroblasts, we used an antibody against fibronectin, a glycoprotein known to be expressed in this cell type (Ibanez et al. 2007). We identified several spindle‐like and oval‐shaped fibronectin‐positive cells localized in the superficial layers of the ON (see Figures 1 and 4).

Using an *mpeg1*‐GFP transgenic *Xenopus* line, we identified a small number of round to oval‐shaped cells located in superficial positions of the ON (see Figures 1 and 4). Given that *mpeg1* is predominantly expressed in macrophage lineages (Ellett et al. 2011; Bayly‐Jones et al. 2020; Ferrero et al. 2020), we classified these cells as macrophages.

**FIGURE 7:**
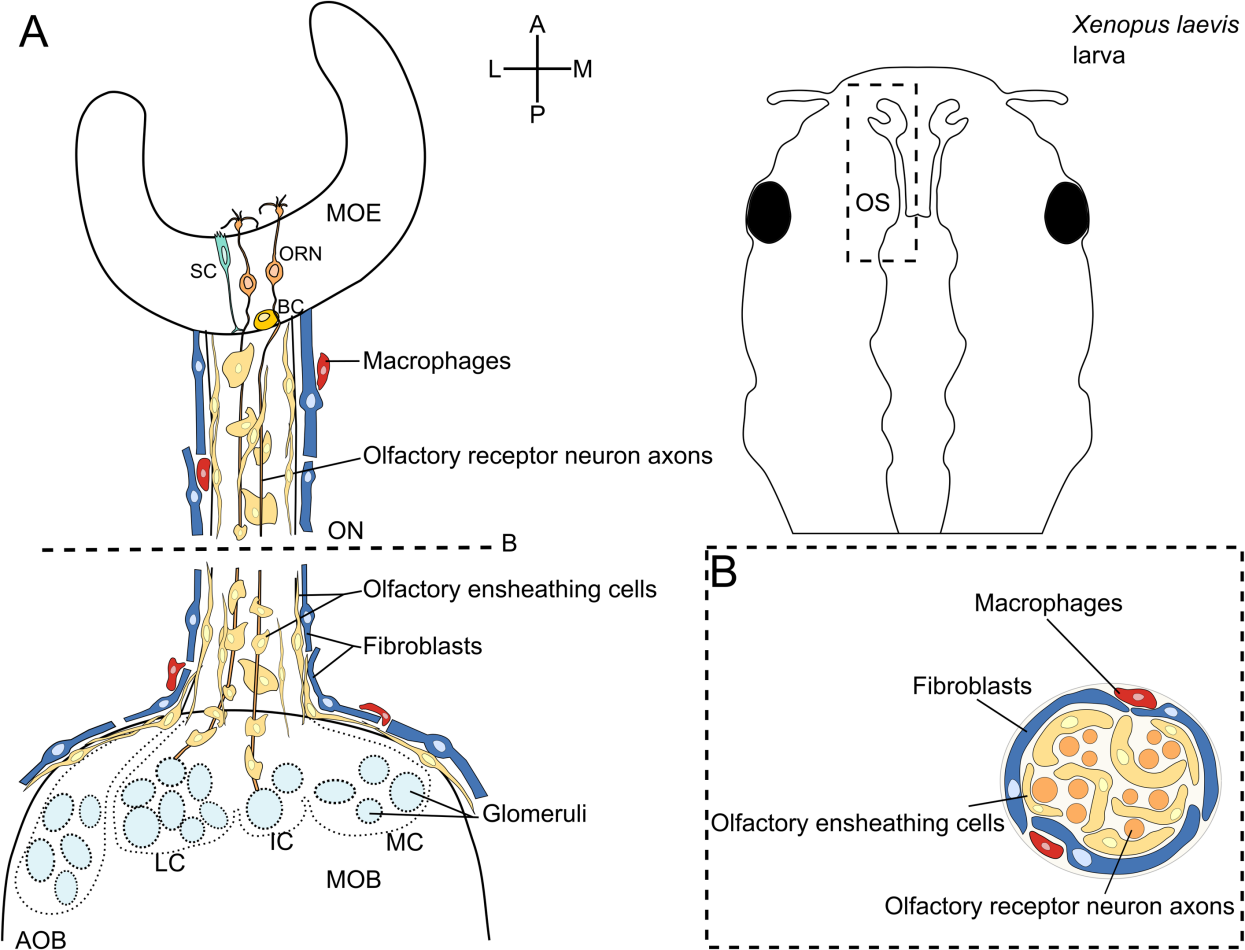
Scheme summarizing the cellular composition of the peripheral olfactory system and olfactory nerve in larval *Xenopus laevis.* (A) The main olfactory epithelium (MOE) contains three main cellular components: olfactory receptor neurons (ORN), non‐neuronal supporting cells (SC), and olfactory stem cells/basal cells (bcs). Olfactory receptor neurons extend their axons through the basal lamina of the OE, the olfactory nerve (ON), and enter the olfactory bulb (OB), where they converge into olfactory glomeruli. In the ON, ORN axons are enwrapped by olfactory ensheathing cells (OECs), which ensheathe several axons into fascicles. The ON is surrounded by fibroblasts, which form a perineurium‐like structure. A small number of macrophages are located outside this perineurium. The rectangle on the schematic of the larva (right‐hand side) indicates the location of the schematic shown on the left‐hand side. (B) Illustration of a cross‐section through the ON showing the location and arrangement of ORN axons, OECs, fibroblasts, and macrophages. A, anterior; AOB, accessory olfactory bulb; BC, basal cell; IC, intermediate cluster; L, lateral; LC, lateral cluster; M, medial; MC, medial cluster; MOB, main olfactory bulb; MOE, main olfactory epithelium; ON, olfactory nerve; ORN, olfactory receptor neuron; OS, olfactory system; P, posterior; SC, supporting cell.

Together, these findings indicate that the identity and distribution of non‐neuronal cells in the ON of larval *X. laevis* are comparable to that found in rodents: OECs surround bundles of ORN axons, fibroblasts form a structure resembling the perineurium, and the number of macrophages on the surface of the ON is relatively low. Our results, however, show that, unlike in rodents, fibroblasts in *Xenopus* do not ensheath nerve fascicles but rather ensheath the whole ON. Also, macrophages are solely located on the surface of the ON and are not present on the surface of individual nerve fascicles. Figure 7 summarizes our findings and renders the cellular composition of the ON in larval *X. laevis* .

### 4.2 Distinct Morphologies of Putative Olfactory Ensheathing Cells in the ON of Larval *X. laevis*

Using antibodies against vimentin, p‐rpS6, and single‐cell electroporations, we identified putative OECs with two morphologies: fusiform bipolar and flat sheet‐like. While putative OECs identified with antibodies against vimentin and p‐rpS6 had a mainly fusiform bipolar morphology (Figures 1, 3, and 4), single‐cell electroporations revealed cells with an additional morphology, i.e., flat sheet‐like. In contrast to fusiform bipolar cells, the flat sheet‐like cells were always located in more superficial layers of the ON. Very likely, OECs with different morphologies fulfil different functions. It would, therefore, be very interesting to investigate if there are any shifts in abundance of either type after ON injury. We will tackle this question in future studies.

It has been shown that vertebrate OECs are a heterogeneous cell population varying in size and morphology (e.g., elongated, flattened and branched), capable of switching between these morphologies (Doucette 1993; Sonigra et al. 1999; van den Pol and Santarelli 2003; Barnett and Chang 2004; Huang et al. 2008, 2024; Pellitteri et al. 2010; Ekberg et al. 2012). Based on this information, we assume that the identified flat sheet‐like cells in the ON of larval *Xenopus* are a type of OEC. This strongly suggests that the morphology of OECs in larval *Xenopus* is generally similar to that in other vertebrates. Nevertheless, further studies are needed to clarify the morphological diversity of OECs in this larval amphibian.

### 4.3 Phosphorylated rpS6 is a Suitable Marker for an Injury‐Mediated Activation of Non‐Neuronal Cells in the ON

Phosphorylation of rpS6, a component of the 40S ribosomal subunit involved in translation, is broadly used as a readout of the mammalian target of rapamycin complex 1 signaling activation and/or as a neuronal activity marker (Meyuhas 2008, 2015; Biever et al. 2015). The phosphorylation of rpS6 was initially noticed in rat liver regeneration (Gressner and Wool 1976), and later it was found that it is promoted by different pharmacological stimuli and enhanced in a variety of physiological and pathophysiological conditions (Wool et al. 1995; Ruvinsky and Meyuhas 2006; Biever et al. 2015). In mouse hepatocytes, growth factors were the primary mechanism responsible for stimulating rpS6 phosphorylation (Pende et al. 2004). The exact biological role of the phosphorylation of rpS6 is, however, still not determined (Biever et al. 2015).

Here, we aimed to monitor the injury‐induced activation of non‐neuronal cells in the peripheral olfactory system of larval *X. laevis* . In initial experiments, we observed that 24 h after unilateral ON‐transection, many non‐neuronal cells were positive for p‐rpS6 within the transected ON and the nerve layer of the ipsilateral OB (Figure 3). Thereby, cells of all previously identified subgroups (*sox10*‐, vimentin‐, fibronectin‐, and *mpeg1*‐positive cells) were activated (Figures 4 and 5) and probably contribute to reparative processes within the ON.

It is well established that OECs are the primary phagocytic cells in the peripheral olfactory system and serve as the primary immune cells of the ON (Su et al. 2013; Nazareth et al. 2015). They bind, produce, and secrete a variety of proteins, such as cytokines/neurotrophins, which act on surrounding cells (Boruch et al. 2001; Woodhall et al. 2001; Nazareth et al. 2015; Barton et al. 2017; Wright et al. 2020). Among others, through the secretion of anti‐inflammatory mediators, OECs can modulate the immune response, potentially reducing inflammation and promoting regeneration by influencing the activity of other immune cells (Denaro et al. 2022; Chen et al. 2025). Also, it has long been known that OECs are involved in the promotion of continuous growth and the targeted pathfinding of ORN‐axons within the ON (Doucette 1993; Key and St John 2002; Ekberg et al. 2012; Ekberg and St John 2014; Barton et al. 2017; Denaro et al. 2022).

Fibroblasts have been shown to produce various cytokines and growth factors, which can activate multiple signaling cascades, including the Ras/Raf/MEK/Erk/MAP kinase and phosphatidylinositol‐3‐kinase (PI3K)/AKT signaling pathways (Meyuhas 2015; Zhang 2017; Plikus et al. 2021). These signaling cascades can lead to the phosphorylation of rpS6 (Meyuhas 2015). Additionally, during tissue development, homeostasis, repair, and disease, fibroblasts secrete extracellular matrix components (Lendahl et al. 2022). These characteristics of fibroblasts may also mediate reparative effects during the regeneration of ON in *X. laevis* .

Macrophages express a diverse array of plasma membrane receptors, enabling them to recognize various endogenous and exogenous ligands (Taylor et al. 2005). Similar to OECs, macrophages are also phagocytic immune cells that have been documented to engulf and degrade apoptotic neuron debris in the CNS (Hume et al. 1983; Napoli and Neumann 2009; Su et al. 2013). However, the ON is almost devoid of macrophages, and ORN axons are not in direct contact with macrophages (Su et al. 2013; Nazareth et al. 2015; Barton et al. 2017; Wright et al. 2020). Our observation that macrophages on the surface of the ON are positive for p‐rpS6 suggests that these cells respond to the secretion of signaling molecules from OECs, fibroblasts, and/or other surrounding cells in response to injury.

Together, our results show that after ON‐transection, phosphorylation of rpS6, i.e., activation, occurs in subgroups of several non‐neuronal cell types of the ON. The activation of these cells is likely involved in modulating the immune response and controlling degenerative and regenerative processes.

### 4.4 Time Course of the Injury‐Mediated Phosphorylation of rpS6 in Non‐Neuronal Cells of the ON

The time course of degeneration and functional regeneration of the olfactory system upon ON‐transection in larval *X. laevis* has been thoroughly investigated (Cervino et al. 2017; Hawkins et al. 2017, 2024). After the transection of the ON, all mature ORNs degenerate, and new ORNs are immediately formed from neuronal stem cells of the OE. One week after transection, the first axons of newly formed ORNs reach the OB and start the synaptic reconnection with OB neurons. Seven weeks after ON‐transection, all connections are functionally reestablished, and after 7 to 9 weeks, characteristic odorant‐induced behavior reappears (Cervino et al. 2017; Hawkins et al. 2017, 2024).

Building on the above findings and our observation that non‐neuronal cells in the ON are activated, i.e., p‐rpS6‐positive, 24 h after ON transection, we set out to analyze the temporal dynamics of this activation. Increased p‐rpS6 levels were detectable within 1 h after transection, peaked after 24 h, and sharply declined after 48 h (see Figure 6). One hour after transection, p‐rpS6‐positive cells were confined to the immediate vicinity of the injury site. Six hours after transection, their number had increased, and they were located along the entire ON up to the rostral portion of the nerve layer of the OB (Figure 6C). After 24 h, p‐rpS6‐positive cells were abundantly distributed throughout the ON and across the entire nerve layer of the OB (Figure 6C). Since it is known that non‐neuronal cells of the ON can secrete a variety of signaling molecules (Boruch et al. 2001; Woodhall et al. 2001; Meyuhas 2015; Nazareth et al. 2015; Barton et al. 2017; Zhang 2017; Wright et al. 2020; Plikus et al. 2021; Hsueh et al. 2024), the observed widespread activation likely results from the diffusion of signaling molecules released by initially activated cells at the injury site, triggering the activation of neighboring cells.

Alterations in the phosphorylation level of rpS6 were also observed in response to traumatic brain injury in rats as early as 30 min to 24 h after injury (Chen et al. 2007). Given that the mammalian target of rapamycin signaling pathways and the phosphorylation of rpS6 promote cellular and neuronal synaptic growth and repair (Ruvinsky and Meyuhas 2006; Chen et al. 2007), it can be speculated that activation of these pathways may also contribute to the regenerative capacity of the olfactory system after nerve injury. It would, therefore, be exciting to study whether rapamycin‐assisted inhibition of the mammalian target of rapamycin‐complex 1 inhibition, which would result in a decreased phosphorylation of rpS6 (Biever et al. 2015), influences the immune response and the overall regenerative capacity of the olfactory system.

## 5 Conclusion

Using transgenic *Xenopus* lines, single‐cell electroporations, and various cell‐type‐specific markers, we identified several non‐neuronal cell types that populate the ON of larval *X. laevis* , including OECs, fibroblasts, and macrophages. Furthermore, we described the spatial localization of these cell types within the ON. Their spatial distribution in *Xenopus* is similar to the distribution of these cell types in the ON of mammalian species. However, unlike in mammals, in *Xenopus*, fibroblasts do not ensheath nerve fascicles but rather ensheath the whole ON. Additionally, unlike in other mammals, macrophages are located on the surface of the ON and are not present within the ON itself. Furthermore, we used the phosphorylation of rpS6 as a proxy for cellular activation after injury. Various non‐neuronal cell types within the ON showed an increased p‐rpS6 signal as early as 1 h to 24 h after ON transection. Morphology, expression of cell type‐specific proteins, location, and injury‐induced activity suggest that the described rpS6‐expressing cells are a mixture of OECs, fibroblasts, and macrophages. Our results show that upon ON transection, the phosphorylation level of rpS6 transiently increases, suggesting that it may be an essential mechanism supporting regenerative processes.

Together, the present study provides the first description of non‐neuronal cells of the ON of larval *X. laevis* and their injury‐induced activation. The results of this study provide the basis for further investigations on how the various non‐neuronal cell types of the ON support regenerative processes in the olfactory system of larval *X. laevis* .

## Author Contributions

**Melina Kahl:** conceptualization, formal analysis, investigation, methodology, validation, visualization, writing – original draft, writing – review and editing. **Lukas Weiss:** conceptualization, formal analysis, investigation, methodology, validation, visualization, writing – original draft, writing – review and editing. **Joshua Walter:** investigation, methodology, validation, visualization, writing – original draft, writing – review and editing. **Thomas Hassenklöver:** conceptualization, data curation, formal analysis, methodology, supervision, validation, visualization, writing – original draft, writing – review and editing. **Ivan Manzini:** conceptualization, funding acquisition, project administration, supervision, validation, visualization, writing – original draft, writing – review and editing.

## Ethics Statement

All animal procedures were performed following the guidelines of laboratory animal research of the Institutional Care and Use Committee of the Justus Liebig University of Gießen (649_M; GI 15/7 Nr. G 89/2017; GI 15/3 Nr. G 15/2018; GI 15/7 Nr. G 2/2019).

## Conflicts of Interest

The authors declare no conflicts of interest.

## Peer Review

The peer review history for this article is available at https://www.webofscience.com/api/gateway/wos/peer‐review/10.1111/ejn.70211.

## Supporting information


**Supporting Figure S1:** Phosphorylated rpS6‐positive olfactory ensheathing cells ensheathe bundles of olfactory receptor neuron axons.
**Supporting Figure S2:** Vimentin‐ and phosphorylated rpS6‐positive cells in the non‐transected olfactory nerve and olfactory bulb.
**Supporting Figure S3:** Co‐localization of HuC/D and phosphorylated rpS6 in the olfactory bulb after transection of the olfactory nerve.

## Data Availability

The data supporting the findings of this study are available at https://doi.org/10.22029/jlupub‐19833.
