# Supplementary material for: Olfactory Nerve Transection Transiently Activates Olfactory Ensheathing Cells in Xenopus laevis Larvae
Source: Eur J Neurosci. 2025 Aug 4;62(3):e70211. doi: 10.1111/ejn.70211 (PMC12320965; doi:10.1111/ejn.70211)
Supplement: Supplementary file 1 — Supporting Figure S1: Phosphorylated rpS6‐positive olfactory ensheathing cells ensheathe bundles of olfactory receptor neuron axons. Supporting Figure S2: Vimentin‐ and phosphorylated rpS6‐positive cells in the non‐transected olfactory nerve and olfactory bulb. Supporting Figure S3: Co‐localization of HuC/D and phosphorylated rpS6 in the olfactory bulb after transection of the olfactory nerve. [file EJN-62-0-s001.pdf]

Olfactory nerve transection transiently activates olfactory ensheathing cells in *Xenopus laevis* larvae

Melina Kahl[1], Lukas Weiss[1,2], Joshua Walter[1], Thomas Hassenklöver[1], and Ivan Manzini[1]*

[1]Institute of Animal Physiology, Department of Animal Physiology and Molecular Biomedicine, Justus-Liebig-University Giessen, 35392, Giessen, Germany

[2]Present address: Department of Ecology and Evolutionary Biology, Princeton University, Princeton, NJ, 08544, USA

*Correspondence: Ivan Manzini (Ivan.Manzini@physzool.bio.uni-giessen.de)

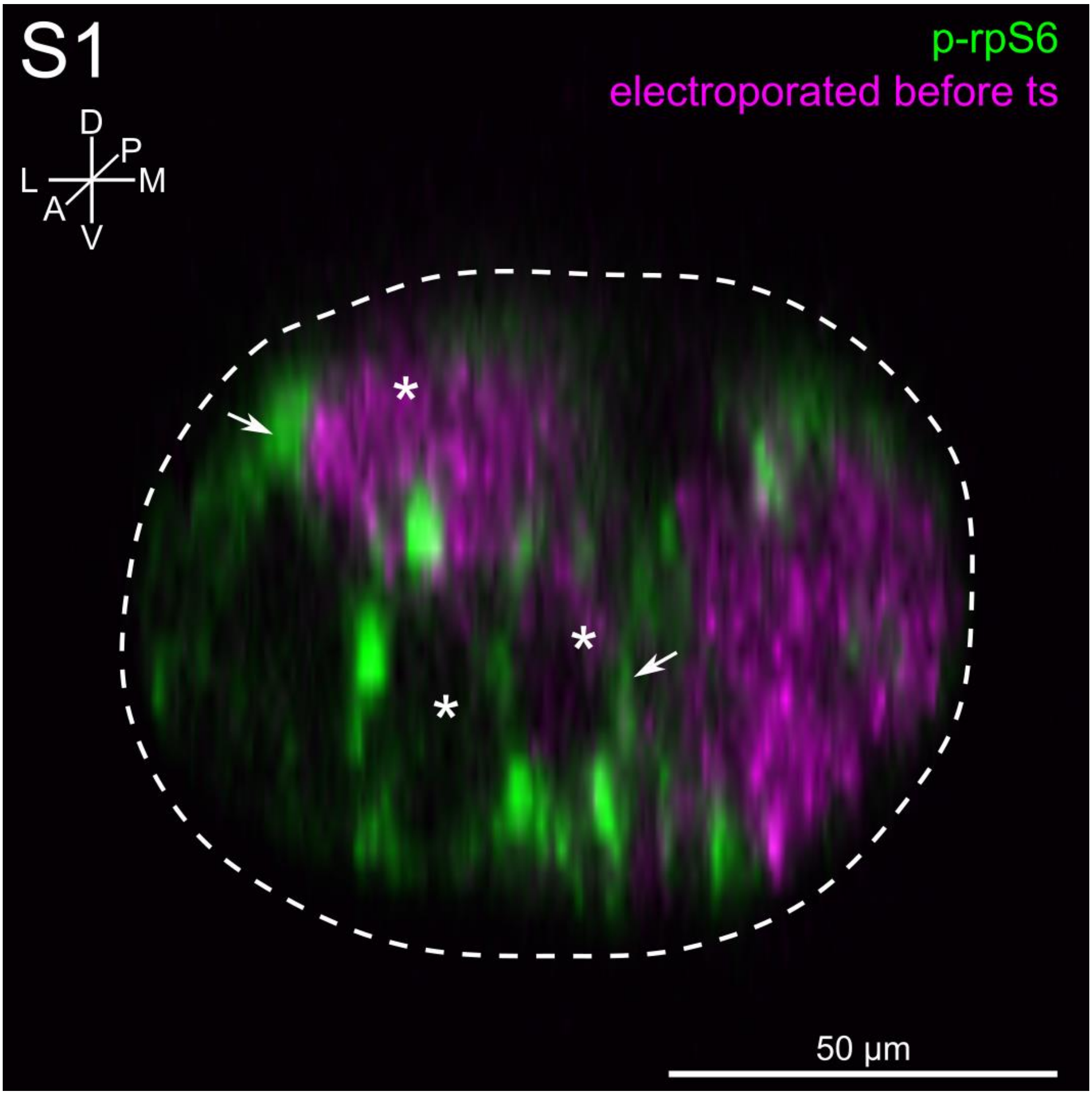

Supporting Figure S1 | Phosphorylated rpS6-positive olfactory ensheathing cells ensheathe bundles of olfactory receptor neuron axons

Transversal view of a transected olfactory nerve (encircled by a white dotted line). Olfactory ensheathing cells (green) stained with an antibody against phosphorylated rpS6 enwrap bundles of olfactory receptor neuron axons (magenta) and form tunnel-like structures (white asterisks). Olfactory receptor neuron axons were labeled by nasal electroporation of fluorescent dextrans.

Abbreviations: A, anterior; D, dorsal; L, lateral; M, medial; P, posterior; V, ventral.

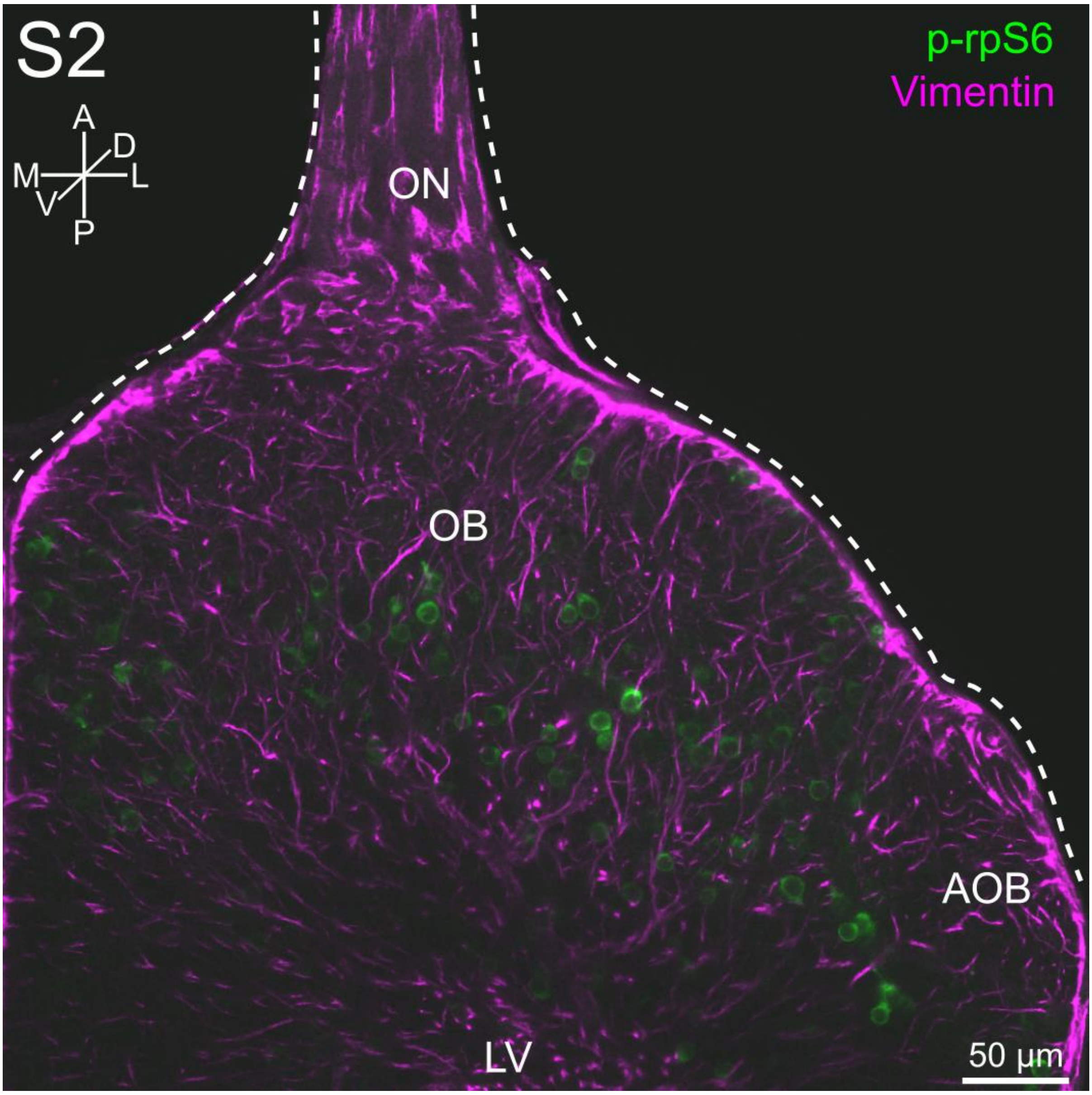

Supporting Figure S2 | Vimentin- and phosphorylated rpS6-positive cells in the non-transected olfactory nerve and olfactory bulb

Vimentin (magenta) was detectable in cells of the olfactory nerve and radial glial cells in the olfactory bulb. Phosphorylated rpS6-positive cells (green) were localized in the glomerular-, mitral cell-, and granule cell layers of the olfactory bulb. Similar results were obtained in all animals investigated (n = 9).

Abbreviations: A, anterior; AOB, accessory olfactory bulb; D, dorsal; L, lateral; LV, lateral ventricle; M, medial; OB, olfactory bulb; ON, olfactory nerve; P, posterior; V, ventral.

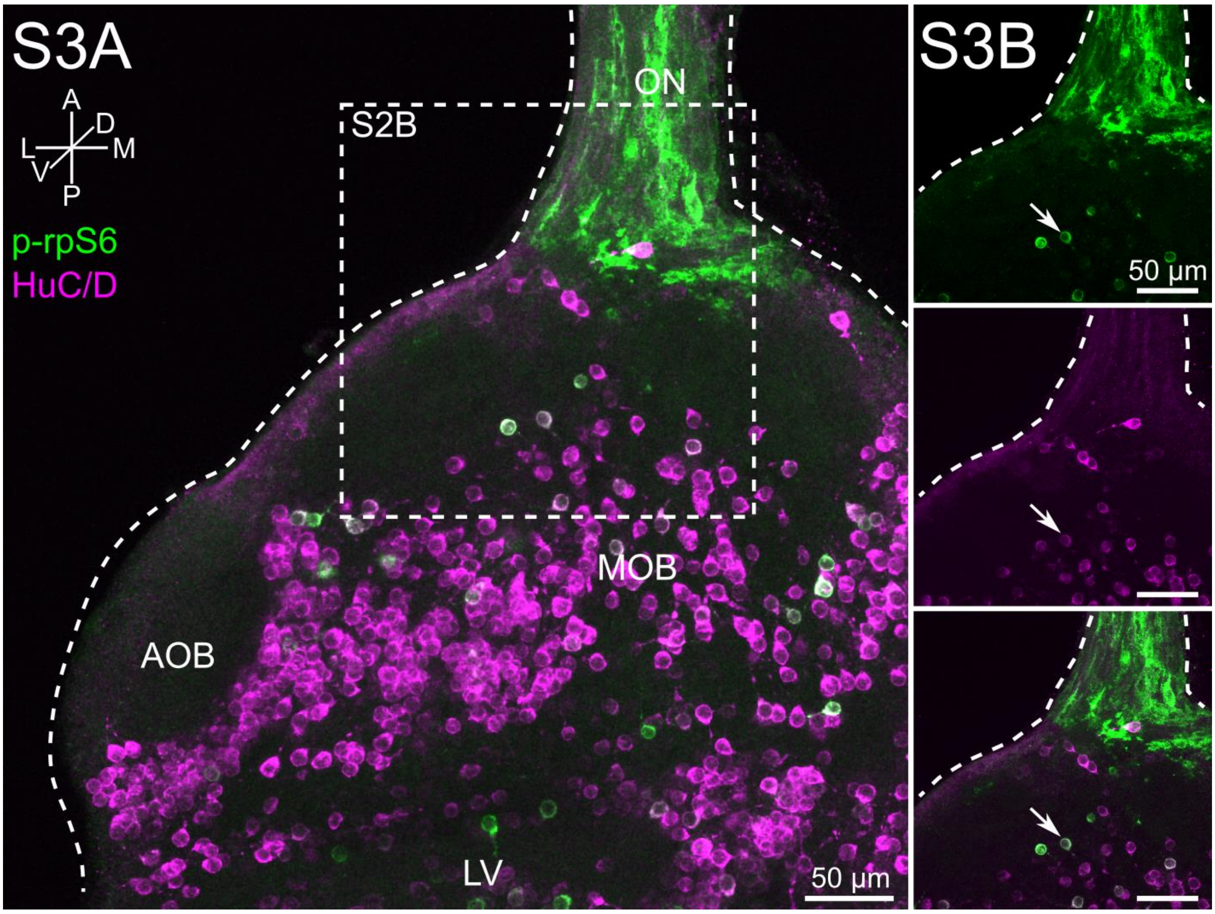

Supporting Figure S3 | Co-localization of HuC/D and phosphorylated rpS6 in the olfactory bulb after transection of the olfactory nerve

(S3A) A subgroup of neurons labeled with an antibody against HuC/D (magenta) in the olfactory bulb are also stained with an antibody against phosphorylated rpS6 (green). A strong staining against phosphorylated rpS6 of non-neuronal cells is present in the olfactory nerve. **(S3B)** Close-ups of the area included in the dotted square in S3A. Upper image: green channel; middle image: magenta channel; lower image: both channels merged. The arrows indicate a double-stained neuron (HuC/D and phosphorylated rpS6). Similar results were obtained in all animals investigated (n = 8).

Abbreviations: A, anterior; AOB, accessory olfactory bulb; D, dorsal; L, lateral; LV, lateral ventricle; M, medial; MOB, main olfactory bulb; ON, olfactory nerve; P, posterior; V, ventral.
